# Supplementary material for: The Systems Biology Research Tool: evolvable open-source software
Source: BMC Syst Biol. 2008 Jun 29;2:55. doi: 10.1186/1752-0509-2-55 (PMC2446383; doi:10.1186/1752-0509-2-55)
Supplement: Additional file 1 — SBRT Archive. An archive of the current version of the Systems Biology Research Tool. [file 1752-0509-2-55-S1.zip › sbrt-1.4.0/doc/developers_guide/api/sbrt/kernel/math/optimization/ProgramSolverStatus.html]

ProgramSolverStatus


|  |  |  |
| --- | --- | --- |
| **Overview** \| **Package** \| **Class** \| **Use** \| **Tree** \| **Deprecated** \| **Index** \| **Help** | |  |
| **PREV CLASS**   **NEXT CLASS** | **FRAMES**    **NO FRAMES**     **All Classes** |  |
| SUMMARY: NESTED \| ENUM CONSTANTS \| FIELD \| METHOD | DETAIL: ENUM CONSTANTS \| FIELD \| METHOD |  |


---


## sbrt.kernel.math.optimization Enum ProgramSolverStatus

```
java.lang.Object
  java.lang.Enum<ProgramSolverStatus>
      sbrt.kernel.math.optimization.ProgramSolverStatus
```

**All Implemented Interfaces:**: java.io.Serializable, java.lang.Comparable<ProgramSolverStatus>

---

``` public enum ProgramSolverStatus extends java.lang.Enum<ProgramSolverStatus> ```

This enum type is used to indicate the status of a program solver.

**Author:**
:   This enum type was written and documented by
    Jeremiah Wright while in the Wagner lab.

---

**Enum Constant Summary**

`BOUNDED`
  
          Used to indicate a bounded solution exists.

`ERROR`
  
          Used to indicate an error has ocurred.

`FEASIBLE`
  
          Used to indicate a feasible solution exists.

`INFEASIBLE`
  
          Used to indicate no feasible solution exists.

`INFEASIBLE_OR_UNBOUNDED`
  
          Used to indicate no feasible solution exists or the solution is unbounded.

`OPTIMAL`
  
          Used to indicate an optimal solution exists.

`UNBOUNDED`
  
          Used to indicate an unbounded solution.

`UNDEFINED`
  
          Used to indicate an undefined problem.

`UNKNOWN`
  
          Used to indicate an unknown problem.


| **Method Summary** | |
| --- | --- |
| `java.lang.String` | `toString()`             Returns an appropriate string representation of this program solver status. |
| `static ProgramSolverStatus` | `valueOf(java.lang.String name)`             Returns the enum constant of this type with the specified name. |
| `static ProgramSolverStatus[]` | `values()`             Returns an array containing the constants of this enum type, in the order they're declared. |

**Methods inherited from class java.lang.Enum**

`clone, compareTo, equals, getDeclaringClass, hashCode, name, ordinal, valueOf`

**Methods inherited from class java.lang.Object**

`finalize, getClass, notify, notifyAll, wait, wait, wait`

**Enum Constant Detail**

### OPTIMAL

```
public static final ProgramSolverStatus OPTIMAL
```

:   Used to indicate an optimal solution exists.

---


### FEASIBLE

```
public static final ProgramSolverStatus FEASIBLE
```

:   Used to indicate a feasible solution exists.

---


### BOUNDED

```
public static final ProgramSolverStatus BOUNDED
```

:   Used to indicate a bounded solution exists.

---


### INFEASIBLE

```
public static final ProgramSolverStatus INFEASIBLE
```

:   Used to indicate no feasible solution exists.

---


### INFEASIBLE\_OR\_UNBOUNDED

```
public static final ProgramSolverStatus INFEASIBLE_OR_UNBOUNDED
```

:   Used to indicate no feasible solution exists or the solution is unbounded.

---


### UNBOUNDED

```
public static final ProgramSolverStatus UNBOUNDED
```

:   Used to indicate an unbounded solution.

---


### UNDEFINED

```
public static final ProgramSolverStatus UNDEFINED
```

:   Used to indicate an undefined problem.

---


### UNKNOWN

```
public static final ProgramSolverStatus UNKNOWN
```

:   Used to indicate an unknown problem.

---


### ERROR

```
public static final ProgramSolverStatus ERROR
```

:   Used to indicate an error has ocurred.


**Method Detail**

### values

```
public static final ProgramSolverStatus[] values()
```

:   Returns an array containing the constants of this enum type, in
    the order they're declared. This method may be used to iterate
    over the constants as follows:

    ```
    for(ProgramSolverStatus c : ProgramSolverStatus.values())
            System.out.println(c);
    ```

    :   **Returns:**: an array containing the constants of this enum type, in the order they're declared

---


### valueOf

```
public static ProgramSolverStatus valueOf(java.lang.String name)
```

:   Returns the enum constant of this type with the specified name.
    The string must match *exactly* an identifier used to declare an
    enum constant in this type. (Extraneous whitespace characters are
    not permitted.)

    :   **Parameters:**: `name` - the name of the enum constant to be returned. **Returns:**: the enum constant with the specified name **Throws:**: `java.lang.IllegalArgumentException` - if this enum type has no constant with the specified name

---


### toString

```
public java.lang.String toString()
```

:   Returns an appropriate string representation
    of this program solver status.

    :   **Overrides:**: `toString` in class `java.lang.Enum<ProgramSolverStatus>`
    :   **Returns:**: an appropriate string representation of this program solver status.


---


|  |  |  |
| --- | --- | --- |
| **Overview** \| **Package** \| **Class** \| **Use** \| **Tree** \| **Deprecated** \| **Index** \| **Help** | |  |
| **PREV CLASS**   **NEXT CLASS** | **FRAMES**    **NO FRAMES**     **All Classes** |  |
| SUMMARY: NESTED \| ENUM CONSTANTS \| FIELD \| METHOD | DETAIL: ENUM CONSTANTS \| FIELD \| METHOD |  |


---
